# Co-Infection by Waterborne Enteric Viruses in Children with Gastroenteritis in Nepal

**DOI:** 10.3390/healthcare7010009

**Published:** 2019-01-13

**Authors:** Sarmila Tandukar, Jeevan B. Sherchand, Surendra Karki, Bikash Malla, Rajani Ghaju Shrestha, Dinesh Bhandari, Ocean Thakali, Eiji Haramoto

**Affiliations:** 1 Department of Natural, Biotic and Social Environment Engineering, University of Yamanashi, Yamanashi 400-8511, Japan; sar1234tan@gmail.com (S.T.); mallabikash@hotmail.com (B.M.); 2 Institute of Medicine, Tribhuvan University, Kathmandu 1524, Nepal; jeevanbsherchand@gmail.com (J.B.S.); me.dinesh43@gmail.com (D.B.); othakali@gmail.com (O.T.); 3 School of Public Health and Community Medicine, University of New South Wales, New South Wales 2052, Australia; s.karki@unsw.edu.au; 4 Interdisciplinary Center for River Basin Environment, University of Yamanashi, Yamanashi 400-8511, Japan; rajani_ghaju12@hotmail.com

**Keywords:** co-infection, enteric viruses, stool testing, drinking water contamination

## Abstract

Enteric viruses are highly contagious and a major cause of waterborne gastroenteritis in children younger than five years of age in developing world. This study examined the prevalence of enteric virus infection in children with gastroenteritis to identify risk factors for co-infections. In total, 107 stool samples were collected from patients with acute gastroenteritis along with samples of their household drinking water and other possible contamination sources, such as food and hand. The presence of major gastroenteritis-causing enteric virus species (group A rotaviruses, enteroviruses, adenoviruses, and noroviruses of genogroup I) in stool and water samples was examined using quantitative polymerase chain reaction. Among the 107 stool samples tested, 103 (96%) samples contained at least one of the four tested enteric viruses, and the combination of group A rotaviruses and enteroviruses was the most common co-infection (52%, *n* = 54/103). At least one viral agent was detected in 16 (16%) of 103 drinking water samples. Identical enteric viruses were detected in both the stool and water samples taken from the same patients in 13% of cases (*n* = 13/103). Group A rotaviruses were most frequently found in children suffering from acute diarrhea. No socio-demographic and clinical factors were associated with the risk of co-infection compared with mono-infection. These less commonly diagnosed viral etiological agents in hospitals are highly prevalent in patients with acute gastroenteritis.

## 1. Introduction

Acute gastroenteritis is one of the leading causes of morbidity and mortality in developing countries such as Nepal [1,2,3,4,5,6]. Usually, gastroenteritis caused by enteric viruses: group A rotaviruses (RVAs), noroviruses (NoVs), astroviruses, and adenoviruses (AdVs) 40 and 41 comprise a significant proportion of gastroenteritis cases in developed as well as in developing countries [7,8]. Enteric viruses are a group of viruses that can cause acute watery diarrhea and are a major threat to human health worldwide [9]. They are usually prevalent in countries with issues of poor hygiene and sanitation [10]. Each year, more than 1.4 million children die as a consequence of waterborne gastroenteritis [11], with a high proportion of mortality caused due to the lack of timely intervention [12,13]. Despite gastroenteritis being easily preventable and treatable, a higher number of children suffer from the clinical infection during their early age [12]. Although numerous drinking water treatment projects have been implemented in part to combat the disease, life-threatening diarrhea is still a major public health issue [14]. The supply of clean and safe drinking water is not sufficient in Nepal [15,16]. More than half of the population of the country rely on groundwater, rivers, spring, and stone spouts [16] that may be contaminated by unplanned urbanization and rapid population growth.

Among the several etiological agents of severe diarrhea, viruses are the most potent cause, as the presence of even a small number of these microorganism is enough to cause gastroenteritis [17]. The presence of these viruses in drinking water sources has been linked to persistent outbreaks of infection [18]. However, water and food are not the only possible sources of transmission. There are several other factors, such as personal hygiene and sanitation, which are associated with increases susceptibility of human towards gastroenteritis. There are not sufficient data reported about the potential sources of contamination for gastroenteritis. Some studies have described predisposing factors related to gastroenteritis [10,12,13,14]. The presence of fecal indicator bacteria, such as total coliforms, *Escherichia coli*, *Klebsiella*, *Enterobacter*, and *Citrobacter*, in the environment indicates fecal pollution that poses a potential risk to the exposed population. For the rapid and simple identification of fecal contamination in different water sources, fecal indicator bacteria have been used traditionally [19].

Occurrence of co-infection by more than one etiological agent is another challenge to efficient treatment [20]. Investigations of gastroenteritis infections are usually based on bacterial and parasitic agents in many hospitals of Nepal. Access to and availability of routine diagnostic tests for enteric viruses is limited in Nepal, which is a challenging issue. Inadequate data and information are available in relation to the viral etiological agents [1,2,3,4,5] that are responsible for causing gastroenteritis. In Nepal, several studies have been done to find out the viral etiological agents of gastroenteritis, identifying rotaviruses as the leading cause of gastroenteritis among children [1,3,5,6]. Evidence of the presence of NoVs [21], AdVs [2], and other enteric viruses also signifies that more studies should be conducted to find out the etiological agent of viral gastroenteritis. Large-scale studies should be conducted to understand the role of multiple interactions and propose evidence-based medical interventions and environmental changes to fight against these pathogens [22]. This information is essential to decrease the cost of treatment and duration of hospital stay for co-infected children. Enteric viruses cause various types of infection in human with diverse symptoms and are usually transmitted through the fecal–oral route. Infected individuals shed huge numbers of virus particles in the environment through their feces, and these enteric viruses are highly active even in adverse environmental condition [23].

Based on this background, this study was carried out to determine the prevalence of infections with RVAs, AdVs, enteroviruses (EVs), and NoVs-GI among young children with gastroenteritis. This study also aimed to assess the co-infection rate of viral pathogens and examine the association between demographics and clinical symptoms on one hand and mono- and co-infections with enteric viruses on the other. Drinking water, food, and hand swab samples were also collected from children to reveal any correlation among feeding habit, hygiene practice of the cases, and gastroenteritis.

## 2. Materials and Methods 

### 2.1. Study Design

The descriptive cross-sectional study was performed twice a year between September 2016 and April 2017. Stool samples were collected from children under 15 years of age suffering from acute gastroenteritis who were enrolled in Kanti Children’s Hospital, Kathmandu, Nepal during the study period. The Kathmandu Valley, the capital city of Nepal, lies in the central development region of Nepal. The population density of the Kathmandu Valley has grown rapidly from 1.11 million in 1991 [24] to 2.53 million in 2011 [25]. In 2011, the sanitation coverage in urban areas of Nepal was 91%, and 98% of households in Kathmandu Valley had toilets in 2012 [26].

### 2.2. Ethical Review

For the enrollment of the acute watery diarrheal children from Kanti Children’s Hospital, ethical approval was taken from the Ethical Review Board of Nepal Health Research Council (reference number 925), Kathmandu, Nepal. An informed written consent from the caretakers of the children was taken and documented in the questionnaire form before stool specimen collection.

### 2.3. Collection and Processing of Fecal Samples

Stool samples were collected with a spatula in a clean, dry, disinfectant-free, wide mouthed screw-capped container. Prior to collection of the stool samples, patients were provided with instructions for sample collection technique and were requested to provide 20 g of stool specimen in the container. A total of 107 stool samples were collected during the study period. Ten percent fecal suspensions were prepared in phosphate-buffered saline and preserved at −25 °C until DNA and RNA extraction. The fecal samples were analyzed for enteric viruses but not for other fecal indicator bacteria and parasites.

### 2.4. Collection and Processing of Hand Swabs, Water, and Food Samples

Beside stool samples, other samples, such as hand swabs (*n* = 97), drinking water (*n* = 103), and food (*n* = 22), were also collected from the enrolled children. During the questionnaire survey, detailed demographic information, socio-economic data, contact number, sanitation, and hygiene data were taken. Hand swabs of the caretakers or children, depending on whether the children were fed by their caretaker or could eat by themselves, were collected. The hand swabs were collected using a BM Fukitool A kit (GSI Creos, Tokyo, Japan) containing 10 mL of phosphate-buffered saline and a cotton swab. The swab was dipped into the buffer. Drinking water and other possible sources of infection such as fruits and vegetables (food sources) were also collected from the household of each patient. Eleven liters of water samples was collected in 1 L sterile, well-labelled, screw-capped plastic bottles. The water samples were de-chlorinated with 1/100 volume of 5000 mg/L sodium thiosulfate. Similarly, 20 g of food sample was also taken from the children’s house in a well-labelled plastic sandwich bag (27.3 × 26.8 × 0.06 cm). All the collected samples were transported to the Public Health Research Laboratory, Institute of Medicine, Tribhuvan University, maintaining the cold chain.

Ten grams of food samples were weighed and washed in 100 mL of normal saline (0.85 g of sodium chloride in 100 mL of sterile de-ionized water) in a plastic sandwich bag. Subsequently, the food samples were rinsed with normal saline, which was then mixed uniformly by shaking the plastic sandwich bag for about 10 min. The final volume obtained from washed food samples was approximately 100 mL. The water, food, and hand swab samples collectedwere first analyzed for fecal indicator bacteria (total coliforms and *E. coli*) by the most probable number (MPN) method using a Colilert reagent (IDEXX Laboratories, Westbrook, CA, USA). For food and hand swabs, *E. coli* concentration values were expressed as MPN/g and MPN/hands, respectively, depending on the sample type.

### 2.5. Concentration of Viruses in Water Samples

Assessment of the concentration of protozoa and viruses in water samples was done using the electronegative membrane–vortex method [27], as described in a previous study [28]. In brief, 10 L of the drinking water was mixed with 100 mL of a 2.5 mol/L MgCl_2_ solution and then passed through a mixed cellulose membrane filter (diameter = 90 mm; pore size = 0.8 µm). The membrane was vigorously vortexed with an elution buffer containing 0.2 g/L Na_4_P_2_O_7_ 10H_2_O, 0.3 g/L C_10_H_13_N_2_O_8_Na_3_ 3H_2_O, and 0.1 mL/L Tween brand polysorbate 80 in a 50 mL plastic tube, followed by centrifugation at 2000× *g* for 10 min at 4 °C. The supernatant was concentrated further using a Centriprep YM-50 ultrafiltration device (Merck Millipore, Burlington, VT, USA) according to the manufacturer’s protocol. 

### 2.6. Detection of Viruses

Two hundred microliters of both viral concentrate and 10% fecal suspension were used to extract viral DNA using a QIAamp DNA Mini Kit (QIAGEN, Hilden, Germany). Viral RNA was also extracted from 140 µL of each viral concentrate and 10% fecal suspension using a QIAamp Viral RNA Mini Kit (QIAGEN).

Extracted RNA was subjected to reverse transcription using the High-Capacity cDNA Reverse Transcription Kit (Thermo Fisher Scientific, Waltham, MA, USA) following the manufacturer’s instruction. AdVs, EVs, NoVs-GI, and RVAs were determined quantitatively using qPCR from water and stool samples. DNA/cDNA (2.5 µL) was mixed with 22.5 µL of a PCR mixture containing 12.5 µL of Probe qPCR Mix (Takara Bio, Kusatsu, Japan), 0.4 pmol/µL each of forward and reverse primers, and 0.2 pmol/µL of a TaqMan probe. Subsequently, PCR tubes containing the mixtures were placed in a Thermal Cycler Dice Real Time System TP800 (Takara Bio) and incubated at 95 °C for 30 s, followed by 45 cycles of 95 °C for 5 s and 58 °C for 30 s for AdVs [29] and NoVs-GI [30], or 60 °C for 60 s for EVs [31,32] and RVAs [33]. Both positive (artificially synthesized plasmid DNA) and negative controls (PCR-grade water) were run together with the samples during qPCR analysis. Ten-fold serial dilutions of the positive control were used to generate the standard curves. Samples whose cycle threshold (Ct) value was greater than 40 were considered negative.

### 2.7. Process Control

To determine if inhibition occurred during qPCR amplification, artificially synthesized plasmid DNA-containing sequences amplified by qPCR assays for chicken parvovirus [34] and porcine teschovirus [35] were used as a process control as described previously [36]. These two viruses were selected because they are not commonly present in human stool and drinking water. In brief, 2.5 µL of cDNA was inoculated with 22.5 µL of a reaction buffer containing 5 × 10^4^ copies of plasmid DNA of chicken parvovirus or porcine teschovirus, 12.5 µL of Probe qPCR Mix, 0.4 pmol/µL each of forward and reverse primers, and 0.2 pmol/µL of a TaqMan probe. The thermal conditions were as follows: 95 °C for 30 s, 45 cycles of 95 °C for 5 s, and 56 °C for 30 s. PCR-grade water was added to a qPCR tube instead of cDNA, which was considered to be a non-inhibition control. The qPCR amplification efficiency was calculated from ratio of the copy number of plasmid DNA in the sample qPCR tube to that in the non-inhibition control tube. The calculated efficiencies for stool and water samples ranged from 55 to 158% and from 65 to 195%, respectively, indicating that there was no inhibition during qPCR.

### 2.8. Statistical Analysis

IBM SPSS Statistics Version 20.0 (IBM Corporation, Armonk, NY, USA) was used to perform the statistical analysis. Differences in percentages between categories were compared using the chi-square test. The factors associated with co-infection were analyzed further using univariable logistic regression, and an odds ratio with a 95% confidence interval (CI) was calculated. The observed odds ratio gives an indication of the risk of co-infection compared to the reference category. A *p* value <0.05 was considered statistically significant. Multiple regression models were not run because of the limited sample size.

## 3. Results

### 3.1. Detection of Enteric Viruses in Stool Samples

Table 1 summarizes the distribution and concentrations of enteric viruses according to age groups. In total, 103 out of 107 samples (96%) were positive for at least one of the four enteric viruses tested. RVAs were detected in 91% of the samples (*n* = 97), EVs were detected in 55% of the samples (*n* = 59), AdVs were detected in 28% of the samples (*n* = 30), and NoVs-GI were found in 5% of the samples (*n* = 5). There was no significant difference in positive percentages between female (*n* = 46/47, 98%) and male children (*n* = 57/60, 95%) (*p* > 0.05). The highest number of samples (50/107, 47%) was from the age group of 0–11 months. In the age groups of 24–35, 36–47, and 48–59 months, positive percentages were 100%. The mean of the total enteric virus concentrations, which was calculated as the arithmetic sum of the four enteric viruses tested, was 6.9 ± 2.1 log copies/g (*n* = 103).

### 3.2. Detection of Enteric Viruses in Drinking Water

Table 2 summarizes the results of detection of enteric viruses in drinking water samples. At least one of the four enteric viruses tested was detected in 16 out of 103 drinking water samples (16%). EVs were detected most frequently in water samples (9/103, 9%), followed by RVAs (5/103, 5%), AdVs (4/103, 4%), and NoVs-GI (4/103, 4%,). Arithmetic mean of the total enteric virus concentrations was 3.9 ± 3.8 log copies/L (*n* = 16).

### 3.3. Co-Occurence of Enteric Viruses

Same set of enteric viruses were analyzed from both stool and water samples. As shown in Table 3, co-occurrence of enteric viruses (68/103, 66%) was found significantly higher than the presence of single enteric viruses (35/103, 34%) (*p* < 0.05). On the basis of the occurrence of single enteric virus data, RVAs were the most frequently detected (33/97, 34%), followed by AdVs (1/30, 3%) and EVs (1/59, 2%). NoVs-GI were detected in combination with other enteric viruses only.

In case of the water samples, AdVs (*n* = 3/4, 75%) were the most frequently detected, followed by EVs (*n* = 6/9, 67%), RVAs (*n* = 3/5, 40%), and NoVs-GI (*n* = 1/4, 25%). The positive percentage of single enteric viruses in the water samples (*n* = 12/16, 75%) was significantly higher than that of multiple enteric viruses (*n* = 4/16, 25%) (*p* < 0.05).

Table 4 shows the combinations of two or more enteric viruses. RVAs and EVs were found together in 33% of the stool samples (*n* = 35/107), RVAs, AdVs, and EVs were found together in 15% of the stool samples (*n* = 16/107), and RVAs and AdVs were found together in 8% of the stool samples (*n* = 9/107). 

### 3.4. Detection of Fecal Indicator Bacteria from Hand Swab and Food Samples

As summarized in Table 5, *E. coli* was detected in 14% (*n* = 14/97) of hand swab samples and 45% (*n* = 10/22) of food samples, with concentrations of 1.9 ± 3.0 log MPN/hands and 2.7 ± 3.9 log MPN/g food, respectively. Total coliforms were observed in 63% (*n* = 61/97) of the hand swab samples and 72% (*n* = 16/22) of the food samples, with a concentration of 1.9 ± 3.5 log MPN/hands and 3.9 ± 4.0 log MPN/g, respectively. 

### 3.5. Relationship between Co-Occurrence of Enteric Viruses and Demographic, Clinical, and Environmental Factors

Table 6 shows the relationship between co-occuring viruses and demographic, clinical, and environmental factors. Odds ratios with 95% CI are presented to show the magnitude and uncertainty of the estimates. Univariable logistic regression showed no significant association between the factors studied and the outcome of co-occurance (*p* > 0.05). Univariable logistic regression suggested that the risk of co-occurring infections of two or more organisms was much higher if food samples from a child’s home contained *E. coli* (*p* = 0.03).

## 4. Discussion

In this study, enteric viruses were found to be a major causative agent of gastroenteritis in children suffering from diarrhea. Our analysis demonstrated an overall prevalence of enteric viruses of 96%, which was much higher than the figures reported in previous studies conducted in Korea (44%) [37], Venezuela (59%) [38], Greece (39%) [39], Brazil (11%) [40], and Germany (59%) [41]. High positive percentage of enteric viruses might be caused by poor hygiene practices and inadequate sanitation infrastructures. The etiological agents for viral gastroenteritis have not been recorded in clinical laboratories of Nepal; therefore, a comparison of prevalence data of viral agents is nearly impossible. However, there have been studies on the prevalence of rotaviruses and other enteric viruses in Nepal within privately funded projects [1,3,4,6,42,43,44,45,46]. These studies showed that children under five years of age frequently suffered from viral gastroenteritis.

RVAs appear to be a major causative agent of gastroenteritis in Nepal, and the prevalence may range from 15 to 45% of the total identified gastroenteritis cases [3,4,6,42,43,44,45]. Similarly, the current study showed a very high prevalence of RVAs (91%), followed by EVs (55%), AdVs (28%), and NoVs-GI (5%) in children with gastroenteritis. The higher detection rates of viral agents may be attributed to the use of highly sensitive qPCR. Previous studies used comparatively less sensitive enzyme-linked immunosorbent assays for the detection of RVAs. In other studies, NoVs have been reported as the second leading cause of viral gastroenteritis in children from Japan [47] and Spain [48], and the occurrence of NoVs in our study also signifies the presence of NoVs in Nepal. 

Additionally, a case–control study conducted at Kanti Children’s Hospital in Maharajgunj, Nepal, also showed 8% of children less than five years old were positive for NoVs [21]. Detection of RVAs was comparatively higher in children suffering from diarrhea due to the extremely high number of viruses shed from infected individuals (possibly as high as 10^11^ viruses/g of stool) [45]. The study also found high concentrations of RVAs in stool, ranging from 4.5 to 10.7 log copies/g. RVAs are highly contagious and persistent and they can survive in the air or water long enough to pose a threat to humans and animals [37]. Therefore, vaccines for RVAs should be part of Nepal’s immunization programs; however, this is currently not the case. Introduction of a safe, effective, and affordable RVA vaccine in Nepal is essential to reduce RVA infections.

The concentrations of enteric viruses in children were comparable across all age groups, which suggests that children under 15 years of age are all equally vulnerable to contracting an enteric virus infection. Moreover, routine evaluation of gastroenteritis patients for the presence of enteric viruses is essential to avoid misdiagnosis and inappropriate treatment. In addition, since breastfeeding and oral rehydration therapy have been proven to reduce the incidence of diarrhea, the increased susceptibility of children to infection during early childhood may be due to poorly developed immunity related to eating and nutritional habits [49]. An increase in outdoor activities and a decrease in maternal care and feeding may also be linked to the increased risk of gastroenteritis in children 24 months old and older [50]. 

Fecal–oral route is a major source of transmission of gastroenteritis. Therefore, this study was conducted to find the presence of causative agents in drinking water, hands, and foods of children with gastroenteritis. Occurrence of *E. coli* and total coliforms from the tested hand swabs and food samples signifies that children had a poor hygienic status, and the above vehicles might be some of the potential sources of infection. This study also showed that chances of multiple infection with enteric viruses was significantly higher in the children who consumed *E. coli*-positive food (*p* < 0.05). To the best of the authors’ knowledge, this is the first study conducted in both hospital and household to identify the potential source of gastroenteritis by taking water samples together with food and hand swabs from each individual gastroenteritis-affected child. Sixteen percent of the water samples tested were positive for at least one of the four viruses considered. This was lower than expected, which might be because of the presence of concentrations of enteric viruses in the water samples below the detection limit of the qPCR. Therefore, large volumes of water samples are frequently required for the detection of enteric viruses, as they are present in very low concentrations in water.

In this study, the majority of children consumed water from tap (47%), followed by jar water (35%). A lower number of children consumed water from tankers (5%), stone spouts (0.9%), and shallow tube wells (0.9%). Although two enteric viruses (RVAs and EVs) were detected in spring water, the rates were not sufficient to predict quality of the entire sources of spring water. The second highest positive percentage of AdVs was found in tanker water. The presence of AdVs in drinking and recreational water have been reported to be the potential sources of infections and viral outbreaks [17]. The presence of all four enteric viruses tested in tap water suggested that the water that is being supplied by the municipality was contaminated. Because of the poor quality of the water supplied by the municipality of the Kathmandu Valley to households, people are likely to prefer relying on private vendors of water from tankers and jar water. However, to date, regulations and oversight for the tanker water suppliers have not been established; therefore, viral contamination may be a risk for those who consume tanker water. The water supplied by jar was labelled as treated; however, detection of fecal indicator bacteria in jar water samples brings into question the quality control methods followed by the suppliers [51]. The presence of high concentrations of fecal indicator bacteria and other enteric pathogens in the municipal water supply indicates that water distributed by the municipality may likely pose a serious threat to human health. Therefore, the government should take a suitable action to reduce further contamination in pipe line water sources. Treated drinking water using chlorine, filtration, and boiling has still not produced a guaranteed supply of water that is safe for drinking. Thus, awareness programs and appropriate hygiene practices are essential to improve the treatment, storage, and transportation of water. Similarly, water supplied by pipe was not provided on a continuous basis; therefore, storage of water in the home is popular in developing countries like Nepal. This might be another possible way of contamination, due to microbial infiltration of poorly maintained systems [14,52].

The occurrence of co-infections (64%) was higher than that of mono-infections (34%) in the children suffering from diarrhea. Combined RVAs and EVs appeared as the most frequent mixed infection, followed by combined RVAs, AdVs, and EVs. The high prevalence of co-infections might be due to the fact that these pathogens share same mode of acquisition through the fecal–oral route, and ultimately susceptibility is increased following a primary infection. Poor sanitation, unsafe drinking water, and other common predisposing factors within developing countries enhances the severity of co-infections. Enteric infections involving a single pathogen are serious, and those involving multiple pathogens are even worse. They have a greater effect on weakening immunity as well as on compromising the nutritional status of the children by displacing water and creating a serious ionic imbalance and multiple other physical malfunctions. 

Univariable logistic regression suggests that there was no significant association between the factors which were considered in this study and co-occurrence (*p* > 0.05) of infective organisms. In our study, the clinical picture of children with co-infection was similar to that of children with mono-infection for all clinical signs taken into examination, particularly dehydration. The presence of *E. coli* in the raw food consumed by children, however, increased the risk of co-infections in children (*p* = 0.04). This may be due to the practice of using river water, sewage, and groundwater for agricultural irrigation in the Kathmandu Valley. Several studies have suggested that these water sources are even not suitable for agricultural irrigation [53,54,55], and it is essential that the government take action to enhance wastewater treatment and monitoring.

In the study, there was an evidence of the presence of the same enteric viruses in both stool and water in 13% of the tested samples (*n* = 13/103). The result obtained was lower than expected, but the collected water was only used for drinking purposes; therefore, theoretically it should be free from any potential microbial contaminant. Six percent of household water samples that were collected from the houses of the children were found positive for RVAs. These data could provide evidence of transmission of enteric viruses from drinking water to the children. Enteric viruses are transmitted to natural bodies of water through either human fecal matter or animal sources. To get more information regarding the viral etiological agents and possible transmission factors in the environment, further studies should be conducted using larger sample size and considering a wider variety of risk factors to identify important barriers to safe water access and to warn the government regarding a safe drinking water policy.

## 5. Conclusions

Enteric viruses were detected in both stool samples and drinking water used by children with gastroenteritis. Positive percentages for at least one of the four enteric viruses were found to be comparable across all age groups. The rate of co-infection of two or more enteric viruses was found to be high in children with gastroenteritis, but their clinical presentation was not more severe than that of children with mono-infection with the same pathogens (*p* > 0.05). RVAs were found to be the most frequently occurring enteric viruses, suggesting they are a major cause of diarrhea in Nepal. The World Health Organization recommends that rotavirus vaccines should be included in all national immunization programs, but they have not yet been included in the national immunization schedule of Nepal. The occurrence of identical genera of enteric viruses in water and in stool samples indicates that drinking water is a contributing factor for gastroenteritis. An increased risk of co-infection associated with any other factor was statistically not significant, while *E. coli*-positive food samples showed significant association with the risk of co-infection. Therefore, more variables should be considered to find out the actual causes of gastroenteritis and coinfection.

## Figures and Tables

**Table 1:** Detection of enteric viruses in stool samples.

Age in Months	No. of Tested Samples (%)	No. of Positive Samples (%)					Total Enteric Virus Concentration (Mean ± SD ^a^) (Log Copies/g)
		EVs	NoVs-GI	RVAs	AdVs	At Least One Virus Detected	
0–11	50	26 (52)	2 (4)	46 (92)	13 (26)	48 (96)	7.2 ± 1.8
12–23	20	9 (45)	1 (5)	19 (95)	5 (25)	19 (95)	7.3 ± 1.9
24–35	10	7 (70)	1 (10)	10 (100)	4 (40)	10 (100)	6.6 ± 1.6
36–47	9	4 (44)	0 (0)	7 (89)	3 (33)	9 (100)	6.9 ± 2.4
48–59	4	3 (75)	0 (0)	3 (75)	0 (0)	4 (100)	7.8 ± 2.2
60–179	14	10 (71)	1 (7)	12 (86)	5 (36)	13 (93)	7.2 ± 2.2
Total	107	59 (55)	5 (5)	97 (91)	30 (28)	103 (96)	6.9 ± 2.1

^a^ SD, standard deviation.

**Table 2:** Detection of enteric viruses in drinking water samples.

Source of Water	No. of Tested Samples	No. of Positive Samples (%)				Total Enteric Virus Concentration (Mean ± SD ^a^) (Log Copies/L)
		EVs	NoVs-GI	RVAs	AdVs	
Shallow tube well	11	1 (9)	0 (0)	1 (9)	1 (9)	4.0 ± 4.6
Jar	36	1 (3)	0 (0)	1 (3)	0 (0)	4.3 ± 3.4
Spring	1	1 (100)	0 (0)	1 (100)	0 (0)	3.0
Stone spout	1	0 (0)	0 (0)	0 (0)	0 (0)	NA ^b^
Tanker	5	0 (0)	2 (40)	0 (0)	1 ()	4.2 ± 3.8
Tap	49	6 (12)	2 (4)	2 (4)	2 (4)	4.5 ± 3.6
**Total**	103	9 (9)	4 (4)	5 (5)	4 (4)	3.9 ± 3.8

^a^ SD, standard deviation. ^b^ NA, not available

**Table 3:** Co-occurrence of enteric viruses in stool and water samples.

Samples	Enteric Viruses	No. of Samples Positive for Enteric Viruses (%)		
		Mono-Infection ^a^/ Single Enteric Virus ^b^	Co-Infection ^a^/ Co-Occurrence ^b^	Total
**Stool**	EVs	1 (2)	58 (98)	59 (100)
	NoVs-GI	0 (0)	5 (100)	5 (100)
	RVAs	33 (34)	64 (66)	97 (100)
	AdVs	1 (3)	29 (97)	30 (100)
	Total	35 (34)	68 (64)	103 (100)
**Water**	EVs	6 (67)	3 (33)	9 (100)
	NoVs-GI	1 (25)	3 (75)	4 (100)
	RVAs	2 (40)	3 (60)	5 (100)
	AdVs	3 (75)	1 (25)	4 (100)
	Total	12 (75)	4 (25)	16 (100)

^a^ Stool sample. ^b^ Water sample

**Table 4:** Combination of co-occurring enteric viruses and their positive percentage in children with gastroenteritis.

Combination of Co-Occurrence of Enteric Viruses	No. of Positive Samples (%) in Stool Samples	No. of Positive Samples (%) in Water Samples
AdVs + EVs	3 (1)	0 (0)
AdVs + EVs + NoVs-GI	1 (1)	0 (0)
RVAs + AdVs	9 (8)	0 (0)
RVAs + AdVs + EVs	16 (15)	0 (0)
RVAs + EVs	35 (33)	1 (1)
RVAs + EVs + NoVs-GI	3 (3)	1 (1)
RVAs + NoVs-GI	1 (1)	0 (0)
RVAs + NoVs-GI + AdVs	0 (0)	1 (1)
EVs + NoVs-GI	0 (0)	1 (1)
Total	68 (64)	4 (4)

**Table 5:** Detection of fecal indicator bacteria in hand swab and food samples.

Fecal Indicator Bacteria	Hand Swab Samples (*n* = 97)		Food Samples (*n* = 22)	
	No. of Positive Samples (%)	Concentration (Mean ± SD ^a^) (Log MPN/Hands)	No. of Positive Samples (%)	Concentration (Mean ± SD ^a^) (Log MPN/g)
*Escherichia coli*	14 (14)	1.9 ± 3.0	10 (45)	2.7 ± 3.9
Total coliforms	61 (63)	1.9 ± 3.5	16 (72)	3.9 ± 4.0

^a^ SD, standard deviation.

**Table 6:** Relationship between co-occurring virus status and demographic, clinical, and environmental factors on the basis of positive percentage of stool samples.

Demographic, Clinical, and Environmental Factors	Categories	No. of Positive Samples (%) ^a^		Odds Ratio (95% CI)	*p* Value
		Mono-Infection (*n* = 35)	Co-Infection (*n* = 68)		
Sex	Male	19 (54)	37 (54)	1.00	0.99
	Female	16 (46)	31 (46)	0.99 (0.44–2.26)	
Age	>12 months	20 (57)	32 (47)	1.00	0.32
	≤12 months	15 (43)	36 (53)	1.50 (0.66–3.43)	
Ethnicity	Other	21 (60)	37 (54)	1.00	0.32
	Ethnicity Janajati	14 (40)	31 (46)	1.26 (0.55–2.87)	
Fever	No	17 (49)	34 (50)	1.00	0.70
	Yes	10 (29)	24 (35)	1.20 (0.47–3.07)	
Abdominal pain	No	9 (26)	14 (21)	1.00	0.37
	Yes	18 (51)	44 (65)	1.60 (0.58–4.27)	
Nausea	No	12 (34)	28 (41)	1.00	0.74
	Yes	15 (43)	30 (44)	0.86 (0.34–2.14)	
Vomiting	No	9 (3)	21 (31)	1.00	0.79
	Yes	18 (51)	37 (54)	0.88 (0.34–2.30)	
Water treatment	No	19 (54)	32 (47)	1.00	0.47
	Yes	14 (40)	32 (47)	1.36 (0.58–3.16)	
Water source	Jar	10 (29)	20 (29)	1.00	0.07
	Tap	19 (54)	28 (41)	0.74 (0.28–1.91)	
	Others ^b^	2 (6)	15 (22)	3.75 (0.71–19.70)	
*E. coli* present in food ^c^	No	7 (78)	3 (30)	1	0.04
	Yes	2 (22)	7 (70)	8.2 (1.03–64.9)	

^a^ The total percentages in each factor group may not be equal to 100% due to missing data. ^b^ Water from the following sources: Shallow tube well, spring, stone spout, and tanker. ^c^ Only nine vegetables samples related to children with single enteric virus occurrence and 10 vegetable samples related to children with co-occurrence were tested for *E. coli* and these were used to calculate the proportion with detected or not detected *E. coli* in the above table.
